# Mechanisms of the Cytotoxic Effect of Selenium Nanoparticles in Different Human Cancer Cell Lines

**DOI:** 10.3390/ijms22157798

**Published:** 2021-07-21

**Authors:** Elena G. Varlamova, Mikhail V. Goltyaev, Valentina N. Mal’tseva, Egor A. Turovsky, Ruslan M. Sarimov, Alexander V. Simakin, Sergey V. Gudkov

**Affiliations:** 1 Federal Research Center “Pushchino Scientific Center for Biological Research of the Russian Academy of Sciences”, Institute of Cell Biophysics of the Russian Academy of Sciences, 3 Institutskaya St., 142290 Pushchino, Russia; 1928lv@mail.ru (E.G.V.); goltayev@mail.ru (M.V.G.); mvn3@mail.ru (V.N.M.); turovsky.84@mail.ru (E.A.T.); 2 Prokhorov General Physics Institute of the Russian Academy of Sciences, 38 Vavilove St., 119991 Moscow, Russia; rusa@kapella.gpi.ru (R.M.S.); avsimakin@gmail.com (A.V.S.)

**Keywords:** selenium nanoparticles, carcinogenesis, oxidative stress, ER stress

## Abstract

In recent decades, studies on the functional features of Se nanoparticles (SeNP) have gained great popularity due to their high biocompatibility, stability, and pronounced selectivity. A large number of works prove the anticarcinogenic effect of SeNP. In this work, the molecular mechanisms regulating the cytotoxic effects of SeNP, obtained by laser ablation, were studied by the example of four human cancer cell lines: A-172 (glioblastoma), Caco-2, (colorectal adenocarcinoma), DU-145 (prostate carcinoma), MCF-7 (breast adenocarcinoma). It was found that SeNP had different concentration-dependent effects on cancer cells of the four studied human lines. SeNP at concentrations of less than 1 μg/mL had no cytotoxic effect on the studied cancer cells, with the exception of the A-172 cell line, for which 0.5 μg/mL SeNP was the minimum concentration affecting its metabolic activity. It was shown that SeNP concentration-dependently caused cancer cell apoptosis, but not necrosis. In addition, it was found that SeNP enhanced the expression of pro-apoptotic genes in almost all cancer cell lines, with the exception of Caco-2 and activated various pathways of adaptive and pro-apoptotic signaling pathways of UPR. Different effects of SeNP on the expression of ER-resident selenoproteins and selenium-containing glutathione peroxidases and thioredoxin reductases, depending on the cell line, were established. In addition, SeNP triggered Ca^2+^ signals in all investigated cancer cell lines. Different sensitivity of cancer cell lines to SeNP can determine the induction of the process of apoptosis in them through regulation of the Ca^2+^ signaling system, mechanisms of ER stress, and activation of various expression patterns of genes encoding pro-apoptotic proteins.

## 1. Introduction

To date, a large amount of evidence has been accumulated for the anticancer activity of selenium-containing compounds of various natures [1,2,3,4,5,6,7]. However, there is no clinically recognized anticancer drug based on selenium due to conflicting results indicating the dual role of this microelement in carcinogenesis [8,9,10]. All Se-containing compounds can be divided into three groups (inorganic and organic nature and nanoparticles), which exhibit anticancer activity mainly due to their direct or indirect antioxidant properties. It is known that cancer cells are characterized by increased production of reactive oxygen species (ROS), but at the same time cope with it [11]. However, since selenoproteins and Se-containing compounds of different nature can behave as both prooxidants and antioxidants depending on the cell type, genotype, and dosage, it is difficult to predict the mechanisms of their action within even one cancer cell line. In this regard, there is no clear picture of concentration-dependent regulation and molecular mechanisms of the cytotoxic effect of specific selenium-containing agents against malignant tumors of various etiologies.

In recent decades, studies of the functional features of Se nanoparticles have gained the greatest popularity due to their high biocompatibility, stability, and pronounced selectivity [12,13,14,15,16]. This allows us to consider them as potentially new therapeutic agents that can function both as drug delivery vehicles and directly serve as anticanceroprotective agents. Methods for obtaining nanosized Se particles are diverse and continue to develop intensively, but two main ones can be distinguished: physical and chemical. Physical methods for the preparation of nanoparticles, which consist of intense thermal or force action on the starting material, seem to be the most promising, since they make it possible to obtain nanoparticles with a cleaner chemical composition. It is believed that zero-valent selenium is bioavailable to humans to a very small extent, but it is known that the reactivity of chemical elements in the form of nanoparticles increases. Currently, one of the widespread methods for the synthesis of impurity-free SeNP is laser ablation.

Within the framework of this work, the molecular mechanisms that regulate the cytotoxic effect of SeNP, obtained by laser ablation, were studied by the example of four human cancer cell lines: A-172 (glioblastoma), Caco-2 (colorectal adenocarcinoma), DU-145 (prostate carcinoma), and MCF-7 (breast adenocarcinoma). We and other authors have repeatedly shown that various Se-containing compounds, including SeNP, are capable of inducing apoptosis in cancer cells by activating various signaling pathways and maintaining prolonged endoplasmic reticulum stress (ER-stress). Therefore, in order to get closer to understanding the molecular mechanisms of SeNP cytotoxicity, a series of experiments was carried out to study the mRNA expression patterns of various signaling apoptosis pathways markers, key participants in the adaptive and pro-apoptotic pathways of the UPR under ER-stress, seven ER-resident selenoproteins and Se-containing glutathione peroxidases and thioredoxin reductases—the main regulators of redox homeostasis of Se in the cell in nature.

In addition, since Ca^2+^ is known to be a secondary mediator in the modulation of many physiological processes in the cell, including oxidative stress and apoptosis, and Se and ER-resident selenoproteins are involved in the regulation of calcium homeostasis, a series of experiments was carried out to study the role of SeNP in the regulation of calcium homeostasis in these cancer cells.

## 2. Results

### 2.1. SeNP Concentration-Dependently Decreased Vitality and Proliferative Properties of Various Human Cancer Cell Lines, but Does Not Affect Normal Cells

The cytotoxicity of SeNP in the studied cells was assessed using two independent methods: determination of cell viability using trypan blue dye, which allows us to selectively stain the nuclei of dead cells, and determination of metabolic activity of cells using a colorimetric MTT test.

**Figure 1:**
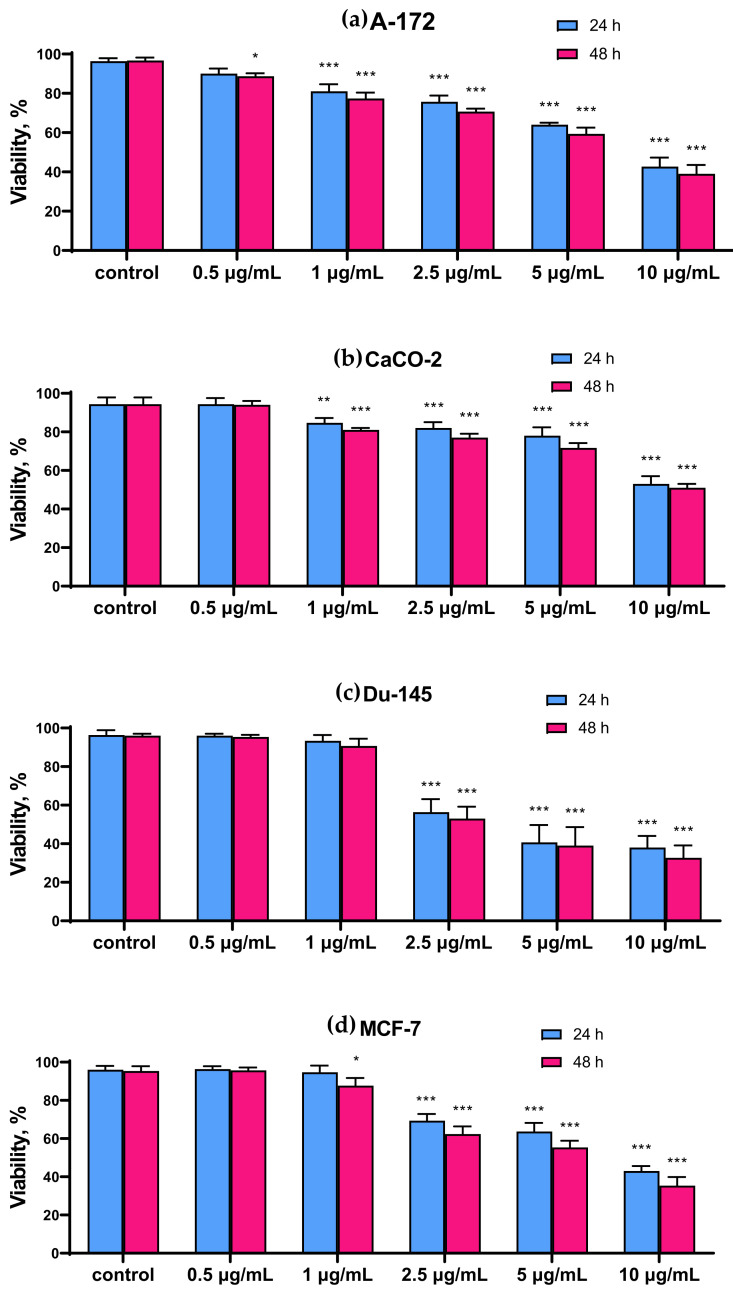

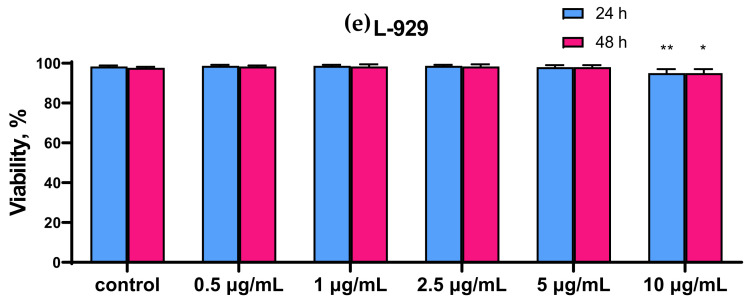
Comparison of the viability of intact cells and SeNP-treated cells for 24 and 48 h (**a**)—A-172; (**b**)—Caco-2; (**c**)—Du-145; (**d**)—MCF-7, (**e**)—L-929. Data are expressed as percentage of control (intact cells; set to 100). Each value is the mean ± SD (*n* ≥ 3, *p* < 0.05). Two-way ANOVA with Sidak’s multiple comparisons test. Comparison with control, *** *p*-level < 0.001, ** *p*-level < 0.01, * *p*-level < 0.05.

It was found that SeNP at concentrations of less than 1 μg/mL had no cytotoxic effect on the studied cancer cells, with the exception of the A-172 cell line, for which 0.5 μg/mL SeNP was the minimum concentration affecting its metabolic activity (Figure 1a). In all studied cancer cell lines, a significant decrease in viability was observed when cells were treated with the highest concentration of SeNP (10 μg/mL). Thus, for MCF-7 and A-172 cells, this concentration of nanoparticles contributed to a decrease in viability by approximately 50%; a similar effect was observed on prostate adenocarcinoma cells (DU-145 cells), however, when they were treated with a half-dose of SeNP (5 μg/mL) (Figure 1a,c,d). Caco-2 cells turned out to be less sensitive to the action of SeNP (Figure 1b). In addition, none of the studied SeNP concentrations appeared to affect the viability of normal (healthy) cells, as which, for example, mouse fibroblasts (line L—929) were selected (Figure 1e). The time of cell treatment with nanoparticles in this experiment was 24 h and 48 h. However, according to the obtained results, no significant differences were found in the viability test. Therefore, all subsequent experiments were carried out before and after 24 h of cancer cell treatment with SeNP.

**Figure 2:**
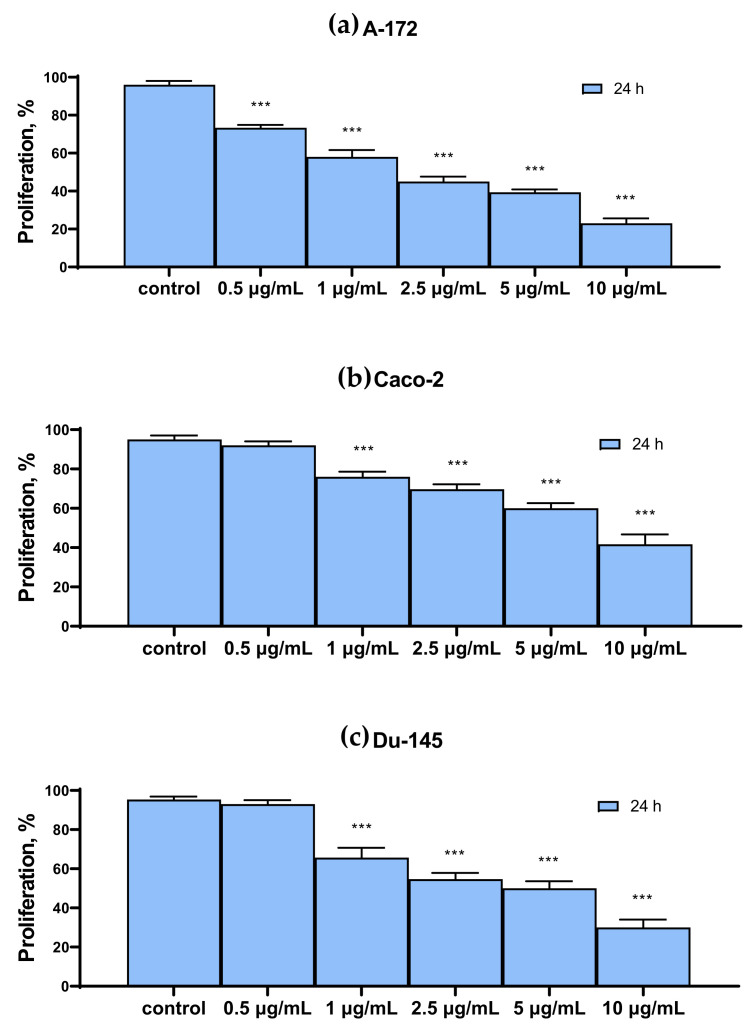

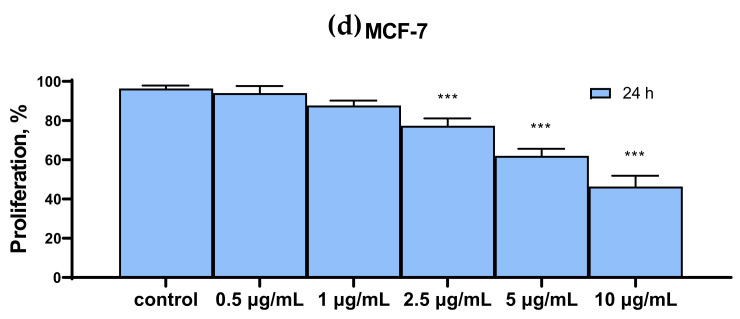
MTT cell proliferation assay of intact cells and SeNP-treated cells for 24 h (**a**)—A-172; (**b**)—Caco-2; (**c**)—Du-145; (**d**)—MCF-7. Data are expressed as percentage of control (intact cells; set to 100). Each value is the mean ± SD (*n* ≥ 3, *p* < 0.05). One-way ANOVA with Sidak’s multiple comparisons test. Comparison with control, *** *p*-level < 0.001.

Figure 2 shows the results of the cytotoxic effect of various SeNP concentrations, obtained by MTT analysis. It can be concluded that the proliferative properties of A-172 and DU-145 cells decreased more significantly compared to the other two Caco-2 and MCF-7. Thus, 24 h treatment of A-172 and DU-145 cells with 10 μg/mL SeNP led to a decrease in their proliferative properties by 70–80% (Figure 2a,c), while for Caco-2 and MCF-7 cells, the figure was approximately 50–60% (Figure 2b,d).

### 2.2. SeNP Concentration-Dependently Caused Cancer Cell Apoptosis, but Not Necrosis

**Figure 3:**
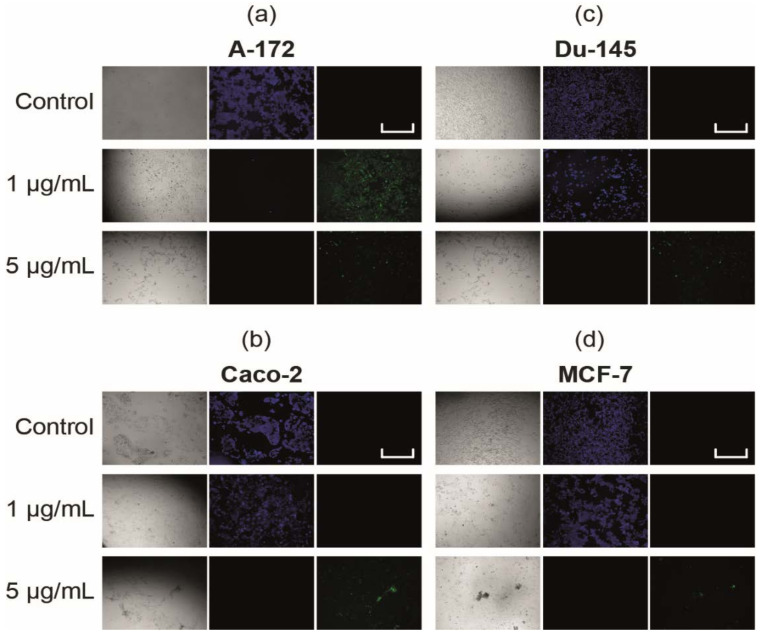
Apoptosis induction in cancer cells by SeNP (**a**)—A-172; (**b**)—Caco-2; (**c**)—Du-145; (**d**)—MCF-7. Cells were incubated with various concentrations of SeNP for 24 h. Apoptotic cells were visualized using the FITC channel (Ex/Em = 490/525 nm). To detect healthy cells, CytoCalcein 450 was used and cells were visualized using the violet channel (Ex/Em = 405/450 nm). Data are expressed as percentage of control (intact cells; set to 100).

Simultaneous monitoring of apoptotic, necrotic, and healthy cells after 24 h of their treatment with 1 μg/mL and 5 μg/mL SeNP using fluorescence microscopy revealed the presence of the apoptotic death of cancer cells of all studied lines when they were treated with 5 μg/mL SeNP (Figure 3), while this was only the case for the A-172 cell line at 1 μg/mL (Figure 3a).

### 2.3. SeNP Enhanced the Expression of Pro-Apoptotic Genes in Almost All Cancer Cell Lines, with the Exception of Caco-2 and Activated Various Pathways of Adaptive and Pro-Apoptotic Signaling Pathways of UPR

In order to understand the cause of apoptotic death of the studied cancer cell lines, we first studied the mRNA expression patterns of the main markers of apoptosis. According to the results of real-time PCR, it can be concluded that when glioblastoma cells are treated with nanoparticles at a concentration of 1 μg/mL, there is a tendency to increase the expression of pro-apoptotic genes: CHOP, GADD34, BIM, and PUMA.

**Figure 4:**
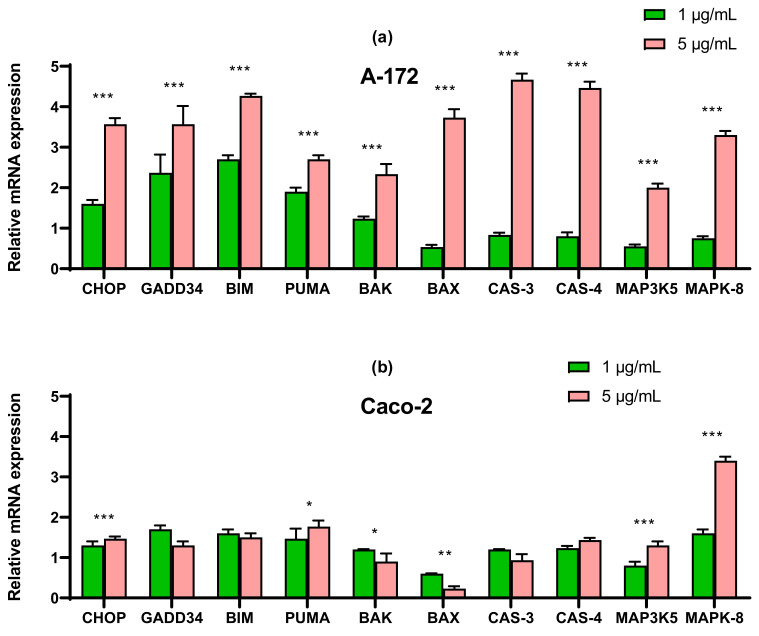

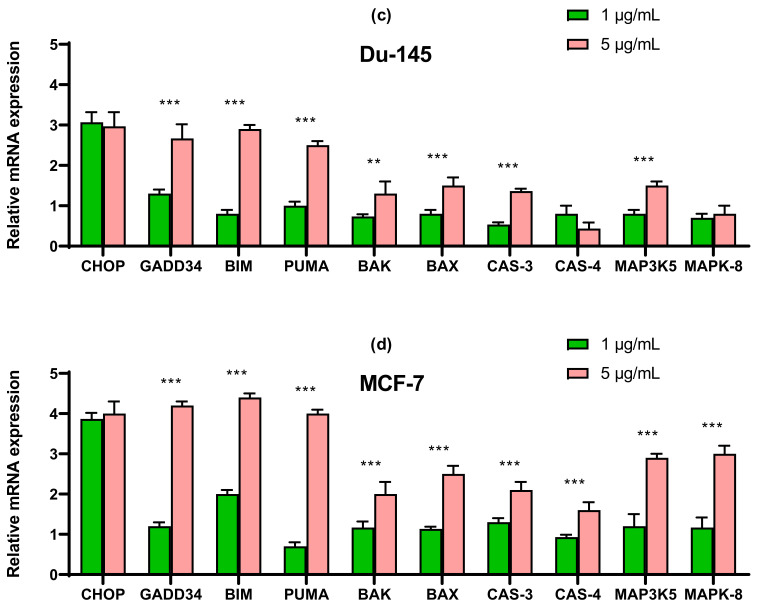
qRT-PCR analysis of the expression of ER stress response in gene (1 and 5 μg/mL SeNP, 24 h)-treated cancer cells (**a**)—A-172; (**b**)—Caco-2; (**c**)—Du-145; (**d**)—MCF-7. Each value is the mean ± SD of at least three independent experiments (*n* ≥ 3, *p* < 0.05). GAPDH was used as an internal control for normalization. Two-way ANOVA with Sidak’s multiple comparisons test. Comparison 1 μg/mL and 5 μg/mL, *** *p*-level < 0.001, ** *p*-level < 0.01, * *p*-level < 0.05.

With an increase in the concentration of SeNP to 5 μg/mL, the expression of all studied genes increased by a factor of 2–4 or more, which indicates the activation of both internal and external pathways of apoptosis (Figure 4a).

In cells Caco-2, SeNP did not significantly affect the expression of pro-apoptotic genes, which explains the absence of apoptosis when cells were treated with 1 μg/mL, whereas when cells were exposed to 5 μg/mL, a significant increase (more than 3 times) in the expression of mitogen-activated kinase 8 was observed (Figure 4b).

In prostate carcinoma cells, an increase in the expression of only four pro-apoptotic genes was observed: p-53-dependent proteins BIM and PUMA, but not BAK and BAX. A threefold increase in the expression of mRNA CHOP and GADD34 was also observed (Figure 4c).

For cells MCF-7, real-time PCR results showed a similar expression pattern to line A-172 for almost all pro-apoptotic genes. There was a significant increase in the expression of CHOP, GADD34, BIM, and PUMA the expression of the other studied genes increased to a lesser extent (Figure 4d).

**Figure 5:**
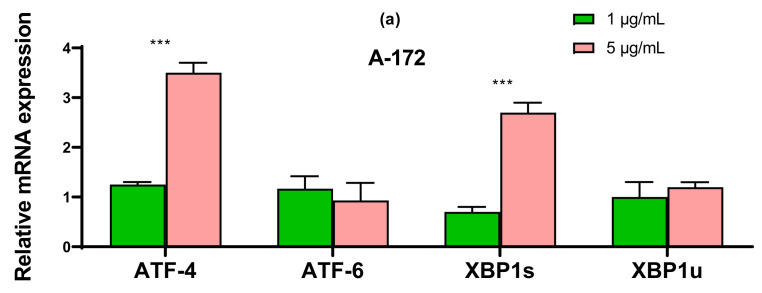

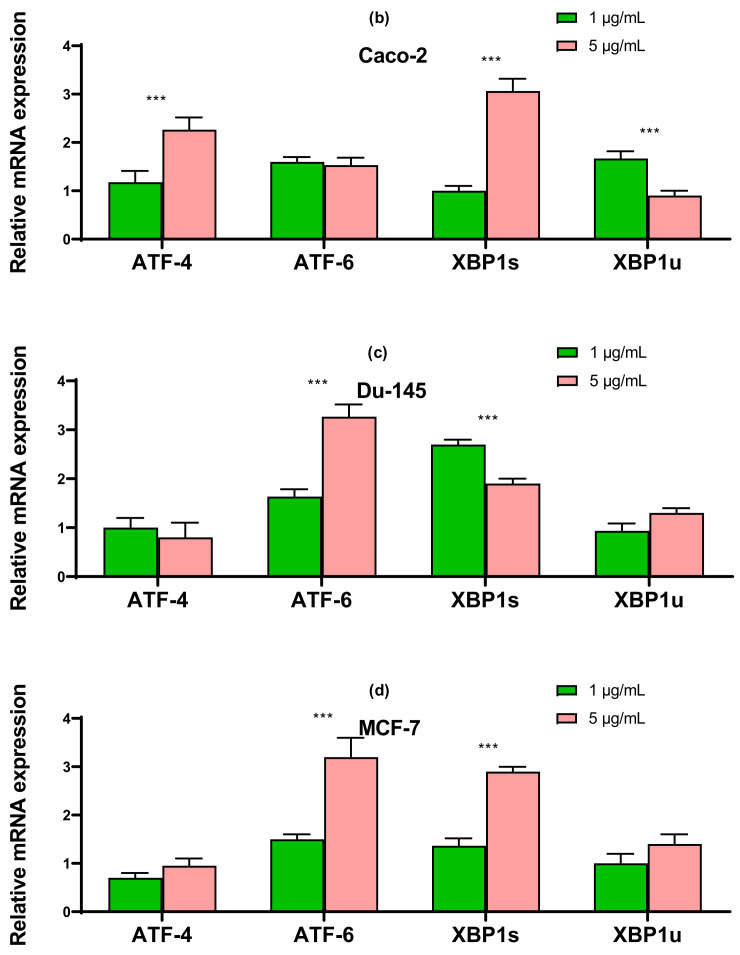
qRT PCR analysis of the expression of ER stress response in gene (1 and 5 μg/mL SeNP, 24 h)-treated cancer cells (**a**)—A-172; (**b**)—Caco-2; (**c**)—Du-145; (**d**)—MCF-7. Each value is the mean ± SD of at least three independent experiments (*n* ≥ 3, *p* < 0.05). GAPDH was used as an internal control for normalization. Two-way ANOVA with Sidak’s multiple comparisons test. Comparison 1 μg/mL and 5 μg/mL, *** *p*-level < 0.001.

**Figure 6:**
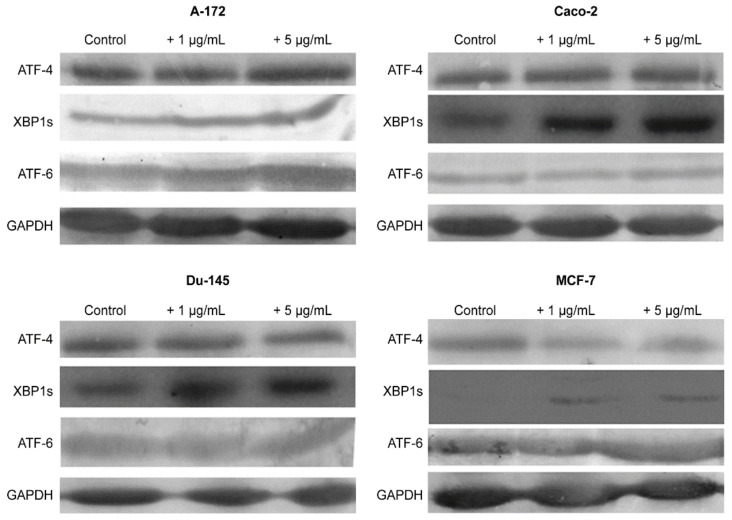
Western blot analysis of ER stress response key markers in various cancer cells after 1 and 5 μg/mL SeNP treatment for 24 h.

**Figure 7:**
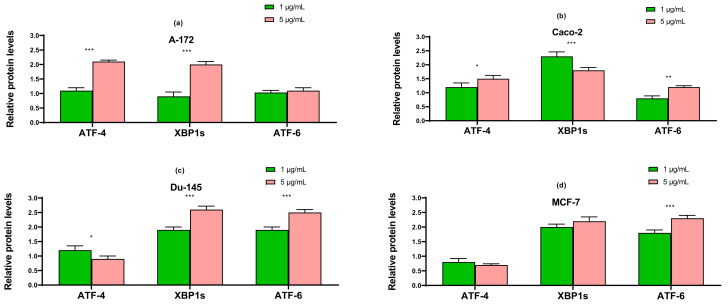
Western blot analysis of ER stress response key markers in various cancer cells (**a**)—A-172; (**b**)—Caco-2; (**c**)—Du-145; (**d**)—MCF-7 after 1 and 5 μg/mL SeNP treatment for 24 h. Data are expressed as percentage of control (intact cells; set to 100). Each value is the mean ± SD (*n* ≥ 3, *p* < 0.05; *t*-test). Two-way ANOVA with Sidak’s multiple comparisons test. Comparison 1 μg/mL and 5 μg/mL, *** *p*-level < 0.001, ** *p*-level < 0.01, * *p*-level < 0.05.

The results obtained using the two approaches of real-time PCR and Western blotting shown in Figure 5, Figure 6 and Figure 7 duplicate others. It can be concluded that SeNP is able to activate in cells A-12 the PERK signaling pathways in A-172 cells, as evidenced by the increased expression of key participants in this ATF-4 pathway. In addition, an increase in the expression of the spliced form XBP1s mRNA may indicate the activation of the IRE1α signaling pathway as well (Figure 5a, Figure 6a and Figure 7a). In cells Caco-2, activation of the signaling pathway mediated by nanoparticles can also be observed (Figure 5b, Figure 6b and Figure 7b). In the other two lines, DU-145 and MCF-7, 5 μg/mL SeNP activated IRE1α and ATF-6 signaling pathways (Figure 5c,d, Figure 6c,d and Figure 7c,d).

### 2.4. SeNP Had Different Effects on the Expression of ER-Resident Selenoproteins and Selenium-Containing Glutathione Peroxidases and Thioredoxin Reductases, Depending on the Cell Line

**Figure 8:**
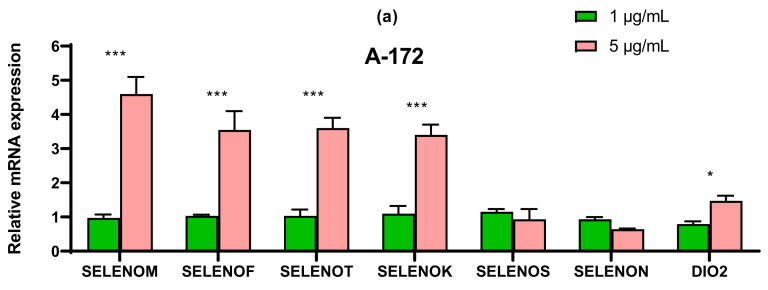

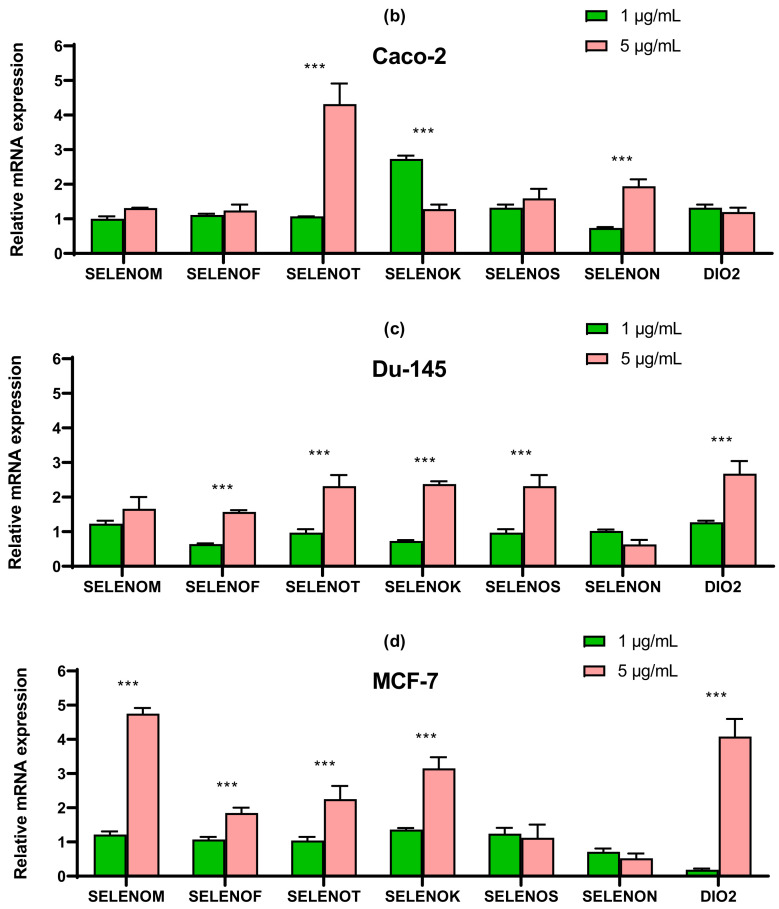
qRT-PCR analysis of the expression of ER resident selenoprotein gene (1 and 5 μg/mL SeNP, 24 h)-treated cancer cells (**a**)—A-172; (**b**)—Caco-2; (**c**)—Du-145; (**d**)—MCF-7. Each value is the mean ± SD of at least three independent experiments (*n* ≥ 3, *p* < 0.05). GAPDH was used as an internal control for normalization. Two-way ANOVA with Sidak’s multiple comparisons test. Comparison 1 μg/mL and 5 μg/mL, *** *p*-level < 0.001, * *p*-level < 0.05.

The SeNP had the greatest effect on the expression of ER resident selenoproteins in cells A-172 and MCF-7. In both cell lines, 5 μg/mL SeNP significantly increased the expression of SELENOM and SELENOK (more than 3–4 times as compared to intact cells) (Figure 8a,d). In glioblastoma cells, an increase in the expression of SELENOF and SELENOT was also observed more than threefold, and in cells MCF-7, a significant increase in the expression of DIO2 mRNA was observed upon treatment of cells with 5 μg/mL SeNP, while 1 μg/mL SeNP, on the contrary, reduced the expression of mRNA of this selenoprotein (Figure 8d).

**Figure 9:**
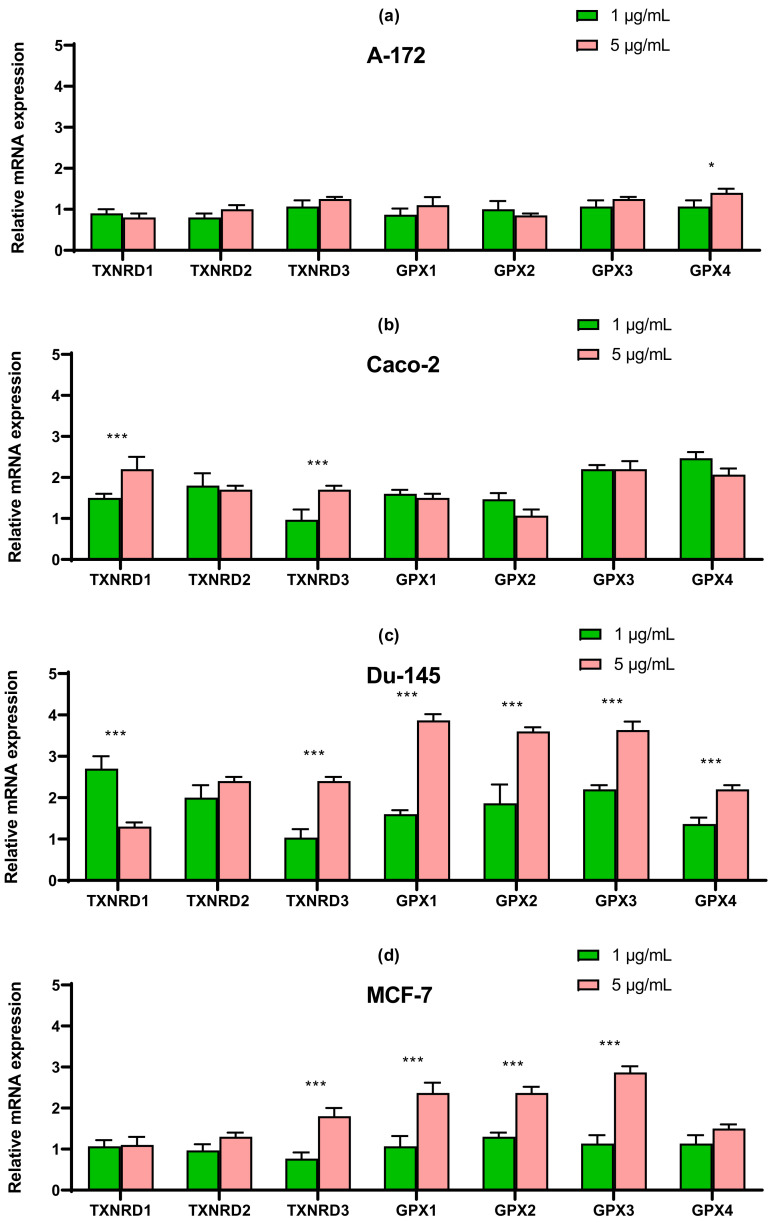
qRT-PCR analysis of the expression of selenium-containing glutathione peroxidase and thioredoxin reductase gene (1 and 5 μg/mL SeNP, 24 h)-treated cancer cells (**a**)—A-172; (**b**)—Caco-2; (**c**)—Du-145; (**d**)—MCF-7. Each value is the mean ± SD of at least three independent experiments (*n* ≥ 3, *p* < 0.05). GAPDH was used as an internal control for normalization. Two-way ANOVA with Sidak’s multiple comparisons test. Comparison 1 μg/mL and 5 μg/mL, *** *p*-level < 0.001, * *p*-level < 0.05.

In line Caco-2, a more than four-fold increase in expression of SELENOT mRNA can be distinguished compared with control (Figure 8b). In prostate carcinoma cells, SeNP did not significantly affect the expression of the seven studied selenoproteins; a twofold increase in the expression of SELENOT, SELENOK, SELENON, and DIO2 can be noted (Figure 8c). Despite this, upon application of SeNP, a significant increase in the expression of almost all selenium-containing glutathione peroxidases and thioredoxin reductases was observed in DU-145 cells (Figure 9c), which is fully explainable by the violation of SeNP-mediated redox homeostasis. On the other hand, in A-172 cells, practically no changes in the mRNA expression of these enzymes were observed (Figure 9a). In the other two lines, 5 μg/mL SeNP had an insignificant effect on the expression, mainly the expression of glutathione peroxidases and TXNRD1 increased almost twofold (Figure 9b,d).

### 2.5. SeNP Triggered Ca^2+^ Signals in All Investigated Cancer Cell Lines

**Figure 10:**
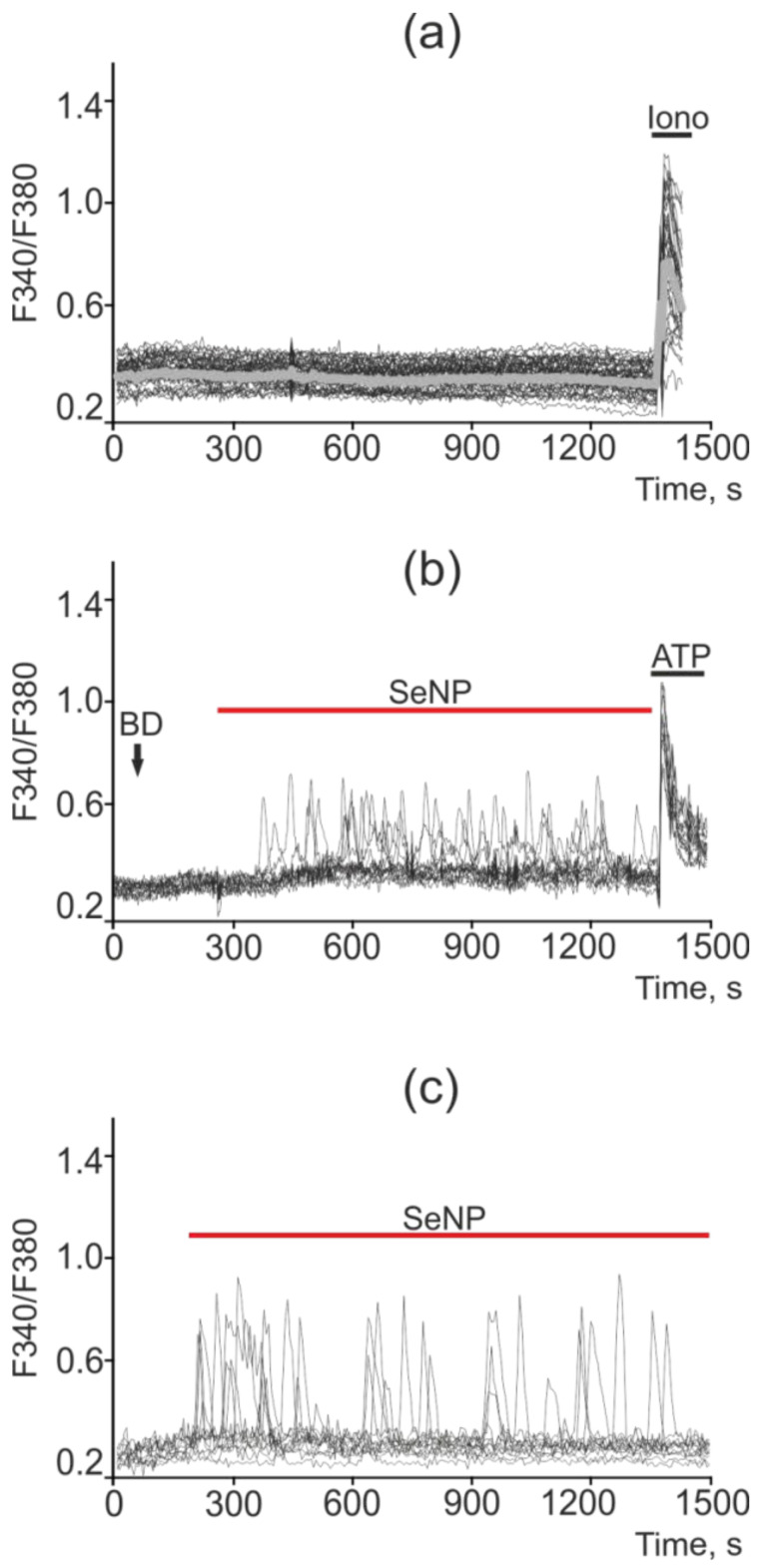
Ca^2+^ signals in control A-172 cells (**a**) and in response to application of 0.5 µg/mL (**b**) and 2.5 µg/mL (**c**) SeNP. BD = application of bi-distilled water equivalent to the amount of SeNP solvent. At the end of the experiment, the Ca^2+^ ionophore, Ionomycin (Iono, 1 µM), or the purinoreceptor activator, ATP (10 µM), was applied to normalize the [Ca^2+^]i signal to the maximum. Typical Ca^2+^ signals of cells are presented.

Changes in the concentration of Ca^2+^ ions in the cytosol ([Ca^2+^]i) regulates most physiological processes in health and carcinogenesis, including the induction of apoptosis. No spontaneous calcium activity was observed in intact A-172 cells (not treated with SeNP) (Figure 10a). Exposure of A-172 cells to 0.5 µg/mL SeNP led to the generation of calcium responses (mainly oscillations) in 70–85% of cells; however, the amplitude of such signals was on average lower and amounted to 0.12 (Figure 10b). In response to the application of 2.5 µg/mL SeNP, Ca^2+^ responses were generated in 30–40% of cells, mainly in the form of oscillations. The average amplitude of the Ca^2+^ signals was 0.25 (Figure 10c).

**Figure 11:**
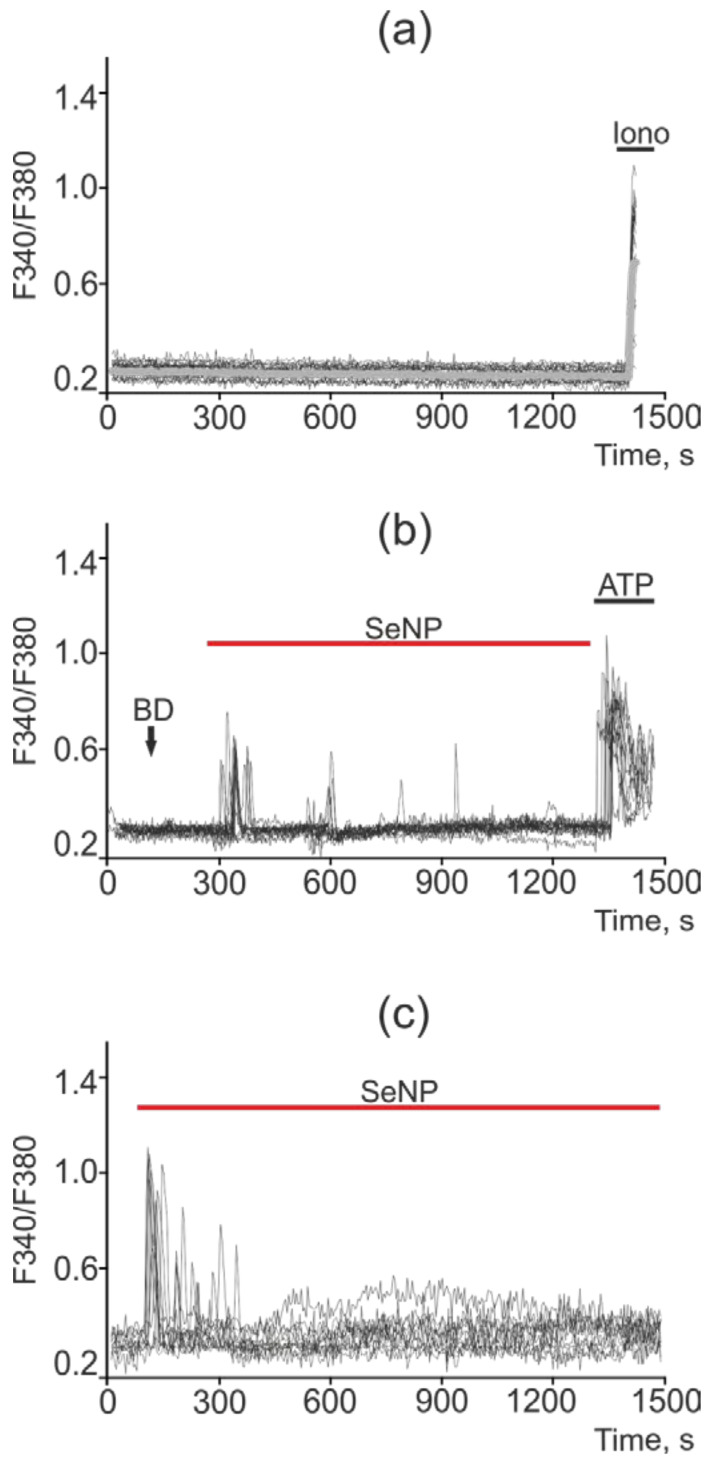
Ca^2+^ signals in control DU-145 cells (**a**) and in response to application of 0.5 µg/mL (**b**) and 2.5 µg/mL (**c**) SeNP. BD = application of bi-distilled water equivalent to the amount of SeNP solvent. At the end of the experiment, the Ca^2+^ ionophore, Ionomycin (Iono, 1 µM), or the purinoreceptor activator, ATP (10 µM), was applied to normalize the [Ca^2+^]i signal to the maximum. Typical Ca^2+^ signals of cells are presented.

In intact DU-145 cells, no spontaneous calcium activity was observed (Figure 11a). In these cells, in response to a lower concentration of SeNP (0.5 μg/mL), an increase in the number of cells with Ca^2+^ responses was observed up to 75%, which was presented mainly in the form of single Ca^2+^ transients and, more rarely, oscillations. In this case, the average amplitude of the Ca^2+^ signals was 0.22 (Figure 11b), whereas in response to the application of 2.5 µg/mL SeNPs, Ca^2+^ responses were generated in 18–25% of cells, mainly in the form of a single pulse or two—three asynchronous pulses. The amplitude of the Ca^2+^ response was comparable to the high-amplitude signal for the application of ionomycin. The average value of the amplitude was 0.37 (Figure 11c).

**Figure 12:**
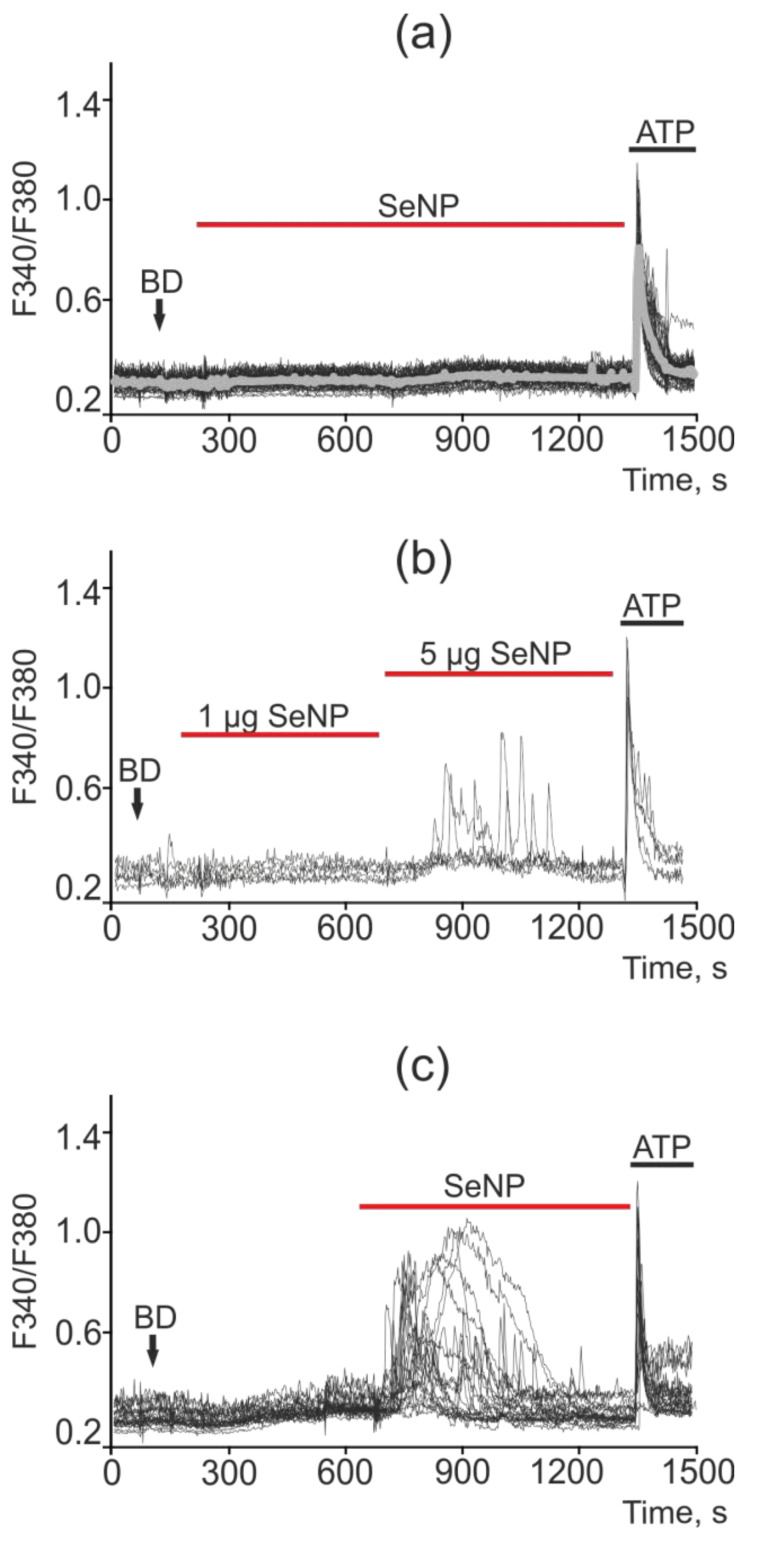
Ca^2+^ signals in control MCF-7 cells (**a**) and in response to application of 1 µg/mL and 5 µg/mL (**b**) and 10 µg/mL (**c**) SeNP. BD = application of bi-distilled water equivalent to the amount of SeNP solvent. At the end of the experiment, the Ca^2+^ ionophore, Ionomycin (Iono, 1 µM), or the purinoreceptor activator, ATP (10 µM), was applied to normalize the [Ca^2+^]i signal to the maximum. Typical Ca^2+^ signals of cells are presented.

The least sensitive to the action of SeNP was the MCF-7 cancer cell line, in which only high concentrations of SeNP (starting from 5 μg/mL) caused the generation of Ca^2+^ signals, as can be seen from Figure 10b,c, upon the application of 1 μg/mL SeNP none of the cells responded. An increase in the SeNP concentration to 5 μg/mL led to a calcium response in only 5 out of 73 cells, i.e., 6.8%. In this case, the cells are alive and functional, since they respond to the application of ATP at the end of the experiment. SeNP at a concentration of 10 μg/mL elicited signals in 100% of cells; moreover, application of ATP at the end of the experiment resulted in high-amplitude signals, which indicates incomplete emptying of the Ca^2+^ pool in the ER; the cells are probably capable of generating Ca^2+^ responses upon subsequent additions of SeNP (Figure 12a–c).

**Figure 13:**
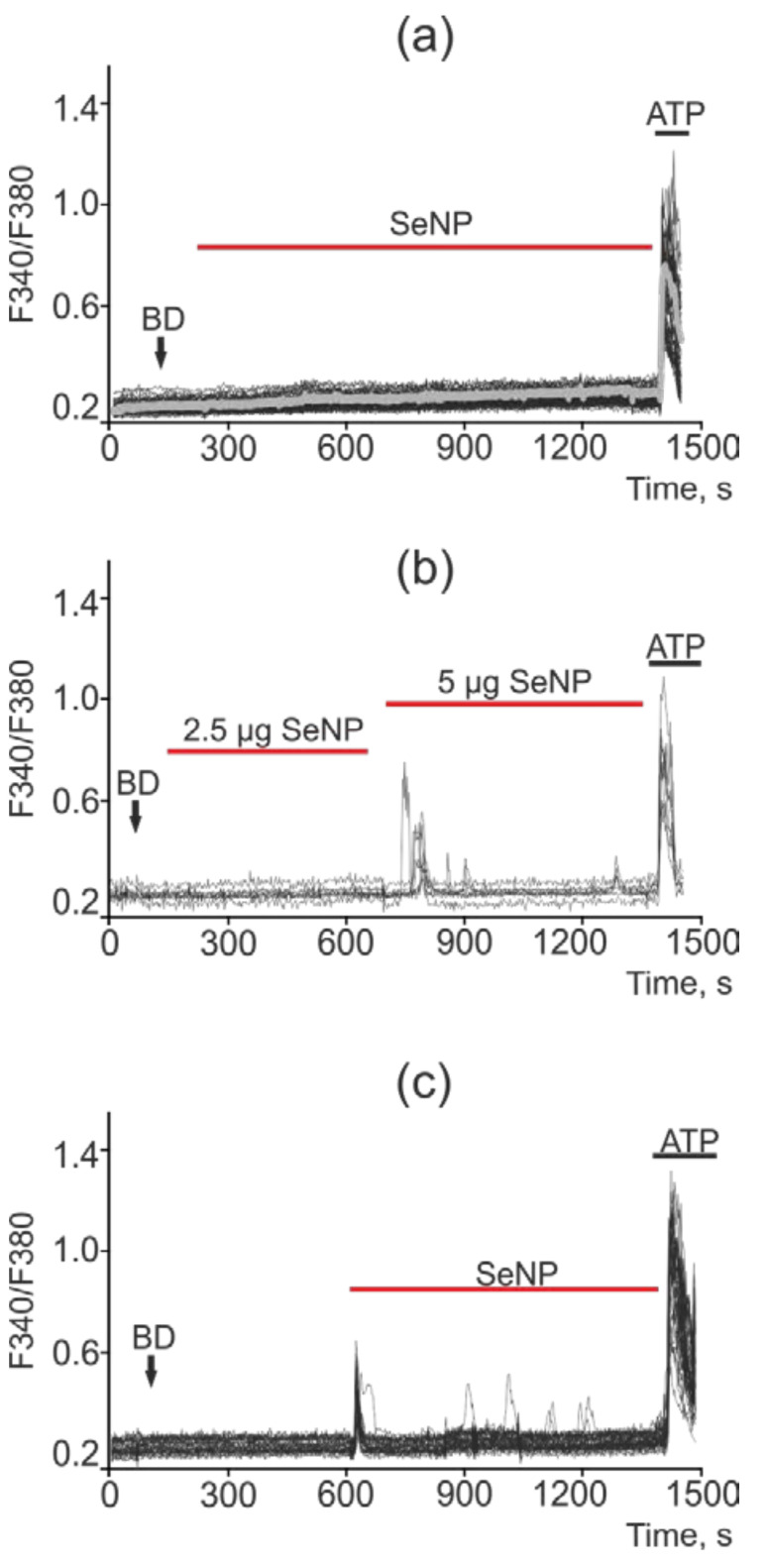
Ca^2+^ signals in Caco-2 cells in response to application of 0.5 µg/mL (**a**), 2.5 µg/mL and 5 µg/mL (**b**) and 10 µg/mL (**c**) SeNP. BD = application of bi-distilled water equivalent to the amount of SeNP solvent. At the end of the experiment, the Ca^2+^ ionophore, Ionomycin (Iono, 1 µM), or the purinoreceptor activator, ATP (10 µM), was applied to normalize the [Ca^2+^]i signal to the maximum. Typical Ca^2+^ signals of cells are presented.

A similar situation was observed in the cells of colorectal adenocarcinoma (Caco-2 line). Thus, most of the cells did not respond to the addition of SeNP at concentrations of 0.5, 1, 2.5, and 5 μg/mL; in the latter case, Ca^2+^ signals were observed only in 5% of cells. An increase in the concentration of SeNP to 10 µg/mL led to Ca^2+^ responses in 30% of cells. In this cell line, Ca^2+^ signals had the form of strictly single impulses, and oscillations were practically absent (Figure 13a–c).

**Figure 14:**
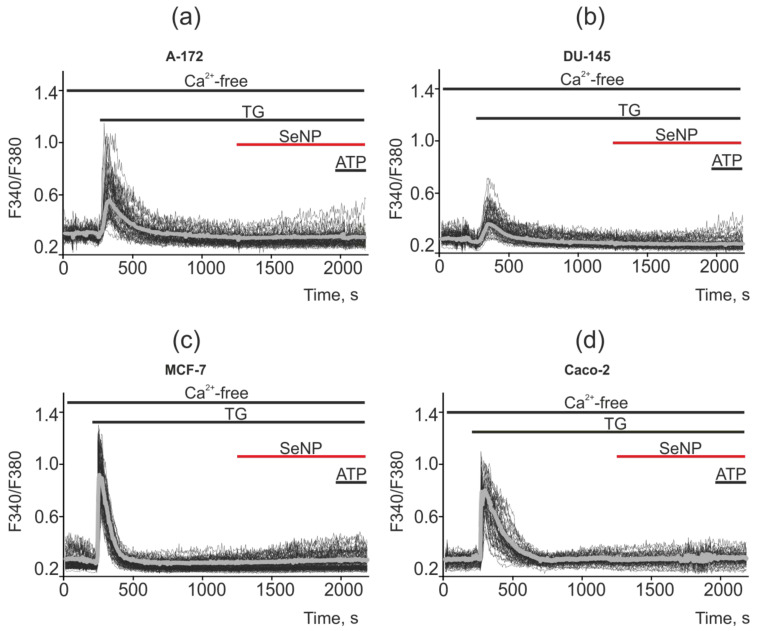
Suppression of Ca^2+^ signals in cancer cells in response to application of SeNP after depletion of the Ca^2+^ ER pool by adding the SERCA inhibitor thapsigargin (TG, 1 µM) in a nominally calcium free medium supplemented with 0.5 mM Ca^2+^ chelator EGTA. (**a**,**b**) application of 0.5 µg/mL SeNP to A-172 and DU-145 cells; (**c**,**d**) application of 10 µg/mL SeNP to MCF-7 and Caco-2 cells. The absence of Ca^2+^ signals for the application of 10 μM ATP indicates a complete absence of Ca^2+^ entering the outside of the cell and mobilization from the ER. Ca^2+^ signals in the field of view of the microscope in one experiment and their average values (gray curves) are presented here.

When measuring the capacity of the thapsigargin-sensitive ER pool in cancer cells, it was shown that in a calcium-free medium (the flow of Ca^2+^ ions is cut off from the outside), the application of thapsigargin (emptying the intracellular Ca^2+^ pool) leads to complete inhibition of Ca^2+^ signals in all cell lines for the application of SeNP. Thus, the generator of Ca^2+^ signals in the studied cancer cells was the thapsigargin-sensitive ER pool, and not the acidic Ca^2+^ pool (lysosomes, caveolae, endovesicles) or mitochondria, etc. (Figure 14a–d). It should be said that, on average, the Ca^2+^ pool of ER is greater in MCF-7 and Caco-2 cells (gray bold curves) compared to A-172 and DU-145, which reflects the large amplitudes of Ca^2+^ signals in response to the TG application.

## 3. Discussion

One of the main goals of nanomedicine is to solve the problems that are usually associated with the use of conventional forms of drugs, in particular, increased safety. At the same time, a large number of works emphasize the unique medical application of selenium nanoparticles (SeNP), which have various therapeutic benefits, including antioxidant, anti-inflammatory, anti-diabetic, and anti-tumor effects [12,13,14,15,16,17,18,19]. It is known that the pharmacological effect and toxicity are highly dependent on the concentration, redox, and type of Se-based compounds [20]. Thus, the antitumor effects of this micronutrient, which have been repeatedly demonstrated, were observed, as a rule, at doses close to toxic. It was found that the SeNP exhibits antitumor activity and low toxicity in comparison with other types of Se. Is is able to scavenge free radicals depending on their size: the lower the size, the better the ability to scavenge free radicals and prevent DNA oxidation. In addition, they showed better bioavailability and biological activity compared to other Se-containing compounds [21].

In our work, nanoparticles had a different concentration-dependent effect on cancer cells of the four studied human lines. There was a direct correlation between the degree of decrease in the viability and proliferative properties of cells with an increase in the concentration of nanoparticles. Glioblastoma cells (A-172 line) turned out to be the most sensitive to the action of nanoparticles: their viability and proliferative properties slightly decreased even when the cells were treated with the lowest studied concentration of SeNP, while this effect was not observed in other cell lines. It is known that glioblastoma is a type of cancer with high mortality due to the inability of chemotherapeutic agents to reach the glioma nucleus. In our work, nanoparticles were not modified with additional ligands required to increase cellular uptake, as was shown in other works [22,23]. In addition, nanoparticles were most effective in reducing the proliferative properties of these cells. It is also worth noting a significant cytotoxic effect on DU-145 cells. In contrast to the other three cancer cell lines, the viability of prostate carcinoma cells decreased by approximately 50% relative to inactive cells after only 24 h treatment of cells with 5 μg/mL of nanoparticles, while a similar effect was characteristic of cells A-172 and MCF-7 when using twice the concentration of nanoparticles (Figure 1). In addition, the proliferative properties of DU-145 cells decreased concentration-dependently with an increase in the SeNP concentrations, similarly to A-172 cells. Given the absence of any significant effect of nanoparticles on these parameters of normal cells, which were taken as mouse fibroblasts (L-929), these results can serve as a serious prerequisite for the creation of modified selenium nanoparticles that have a strong antitumor effect against glioblastoma and prostate adenocarcinoma and do not have a toxic effect on healthy cells. Of course, the L-929 cell line is far from a suitable example of negative control of the SeNP cytotoxic effect; therefore, similar experiments should be carried out on healthy cells surrounding each specific tumor using more relevant models. However, it is nevertheless necessary to emphasize the absence of the cytotoxic effect of even sufficiently high concentrations of SeNP on the viability of non-cancerous cells. In our experiments, there was some selectivity and specificity of the action of nanoparticles.

To elucidate the molecular mechanisms of regulation of the cytotoxic effect of SeNP in these cancer cell lines, a series of experiments was carried out to study the patterns of gene-marker mRNA expression of various apoptosis signaling pathways, key participants in the adaptive and pro-apoptosis pathways of UPR under ER-stress conditions.

Increased expression of a group of genes belonging to the BCL-2 family in A-172 and MCF-7 cells most likely implies activation of the internal mitochondrial apoptosis pathway [24,25]. It is known that these pro-apoptotic proteins are capable of increasing the permeability of the mitochondrial membrane and causing the release of cytochrome C into the cytoplasm. As a result of these sequential processes, the expression of caspase 3 is enhanced, which was also observed in these cancer lines after their treatment with 5 µg/mL SeNP (Figure 4a,c). In addition, in these cell lines, especially A-172, there was an increase in the expression of caspase-4, which is known to be involved in modulating the intrinsic ER-mediated pathway of apoptosis [26,27,28,29].

Additionally, 5 µg/mL SeNP contributed to the enhancement of the expression of mitogen-activated kinases in A-172, Caco-2, and MCF-7 cells (Figure 4a,d). MAP3K5-cytosolic serine/threonine protein kinase, of the MAP3K family, activates JNK and p38 Raf kinases in response to a range of stress signals, including oxidative stress, endoplasmic reticulum stress, and calcium elevation [30]. Thus, it can be assumed that in these cell lines, nanoselenium is able to activate the external signaling pathway of apoptosis, although these data require additional confirmation.

An increase in the expression of the CHOP may also indicate the activation of the mitochondrial signaling pathway of apoptosis. It is known that CHOP regulates the expression of BIM during ER-stress, which promotes translocation of BAX into mitochondria. In addition, it is known that CHOP is activated as a result of triggering the PERK-signaling pathway, which we have shown for line A-172 (Figure 4a) [31,32], and also enhances the expression of pro-apoptotic genes GADD34 and PUMA. In addition, the ATF6-UPR pathway also contributes to the activation of CHOP, as well as its post-translational stimulation with p38 MAPK-kinase [33], as we have shown on cells DU-145 and MCF-7. In addition, in all studied cell lines, an increase in the expression of the spliced form XBP1 was observed (Figure 4, Figure 5, Figure 6 and Figure 7), which may indicate the activation of IRE1α UPR-signaling pathway. Simultaneous activation of both signaling pathways IRE1α and ATF-6 in cells DU-145 and MCF-7 may once again confirm their close relationship, since it is known that ATF-6 affects its target genes in combination with XBP1 [34,35] and increases the expression of XBP1 itself, maintaining IRE1α as a substrate [36].

To date, it is known that in mammals seven selenoproteins are localized in the ER: SELENOM, SELENOF, SELENOT, SELENOK, SELENOS, SELENON, and DIO2. Localized in this organelle, these selenoproteins are involved in the processes occurring in it, the most common of which are participation in protein degradation, in the regulation of ER stress and redox metabolism. The selenoproteins of the ER, according to their structural features, are classified by families. Thus, SELENOM, SELENOF, and SELENOT belong to the family of proteins with thioredoxin-like folding, while SELENOK and SELENOS belong to the family of type III transmembrane proteins [1,2,3,4,5,6,7,8,9,10].

In A-172 and MCF-7 cell lines, 5 μg/mL SeNP significantly increased the expression of SELENOM and SELENOK (more than 3–4 times as compared to intact cells) (Figure 8a,d). This is quite understandable by the fact that SELENOM and SELENOK quite actively respond to changes in the redox status in cells, especially under the conditions of ER stress caused by selenium-containing inducers, which has been demonstrated repeatedly by us and other authors. The known functions of these selenoproteins are very diverse. SELENOM has a thioredoxin-like folding and carries a conservative CXXU motif in the catalytic center (where C is cysteine, X is any two amino acids, U is selenocysteine). [37]. It is known that SELENOM is most sensitive to Se deficiency in the brain, can serve as a molecular biomarker of selenium status in this organ, and is involved in the regulation of human neurogenerative diseases [38]. Similar results have been shown in HT22 hippocampal cells and C8—D1A cerebellar cells: stable overexpression of SELENOM prevents oxidative cell damage caused by hydrogen peroxide [39]. On the contrary, knockdown of the selm gene in rats led to an increase in glutathione peroxidase and thioredoxin-reductase activities in the brain, liver, lungs and, to a lesser extent, in the kidneys and heart. Perhaps this was one of the reasons for the absence of changes in the expression of thioredoxin reductases and glutathione peroxidases in glioblastoma cells, in which a significant increase in SELENOM expression was observed (almost 5 times compared to the control) (Figure 8a). In addition, overexpression of SELENOM in HT22 and C8-D1A cells increases the concentration of cytosolic calcium in response to oxidative stress, and possibly, participates in the regulation of apoptosis by blocking or delaying it [40]. This was shown in our experiments, as concentrations as low as 0.5 μg/mL SeNP caused calcium responses in 70–80% of glioblastoma cells (Figure 10a).

It is known that SELENOK is involved in the regulation of the ERAD system (ER-associated degradation) of many misfolded proteins, as well as components of the OST complex, and is involved in maintaining ER homeostasis [41,42]. In a model of SELENOK gene knockout mice, it was shown that it was involved in the transfer of Ca^2+^ ions in immune cells. However, due to the absence of canonical motifs of Ca^2+^ binding domains (EF-hands repeats, cadherin repeats, etc.) in the primary structure of SELENOK, this selenoprotein is most likely involved in the regulation of calcium ion transport indirectly by interacting with signaling molecules, other ER-membrane proteins, or proteins of the cytoskeleton [43,44,45,46]. In addition, SELENOK possesses peroxidase activity and is capable of reducing harmful hydrophobic substrates such as, for example, phospholipid hydroperoxides, and therefore it is assumed that this protein is involved in the restoration of the membrane bilipid layer.

In addition, a significant increase in SELENOF was observed in A-172 cells (Figure 8a). It is known that SELENOM and SELENOF are homologues not only in different species, but also in classes of organisms. It was found that both proteins are thiol disulfide oxidoreductases with protein disulfide isomerase activity [37,47]. To date, the role of SEP15 in the regulation of ER stress has been well studied, but the mechanism of this regulation largely depends on the inducer that induces ER stress. It was shown that the sep15 gene silencing in CT-26 intestinal cancer cells led to an increase in the expression of the ccnb1ip1 gene, which encodes a protein that functions as ubiquitin ligase and is able to interact with cyclin B, promoting its degradation. Thus, this protein is involved in the regulation of the cell cycle at the G2-M stage [48] and also affects the processes of cell migration and metastasis [49].

In A-172 and Caco-2 cells (Figure 8a,b), there was also an increase in the expression of SELENOT mRNA, which plays an important role in embryogenesis, and participates in the processing of ER proteins and ensuring ER redox homeostasis. It has been shown that SELENOT interacts with keratincyte-associated protein 2 (KCP 2), which is a subunit of the oliosaccharyltransferase protein complex (OST complex) involved in the N-glycosylation of proteins, in particular endogenous glycoproteins. In addition, this selenoprotein interacts with other subunits of the A-type OST-complex, a decrease in SELENOT expression leads to disruption of the N-glycosylation of pro-opiomelanocortin-prohormone synthesized by corticotropic cells of the anterior pituitary gland and melanotropic cells of the middle lobe of the pituitary gland [50]. It was shown that with a decrease in SELENOT activity in cells, proteins with incorrect folding can accumulate, which can violate PACAP-induced mobilization of Ca2+ from extra- and intracellular sources [51]. Using Ca^2+^ -micro-fluormetric analysis, it was shown that overexpression of SELENOT in PC12 cells increases the level of intracellular calcium, and gene silencing of this selenoprotein, on the contrary, suppresses it, which disrupts the PACAP-induced mobilization of Ca^2+^ from extra- and intracellular sources. It is possible that SELENOT interacts directly or indirectly with thiol groups and/or glycosylated sites of intracellular calcium channels and pumps and regulates their activity through a redox mechanism.

In addition, in DU-145 and MCF-7 cells, an increase in the expression of DIO2 was recorded in response to the application of 5 μg/mL SeNP (Figure 8c,d). This enzyme is involved in the activation of thyroid hormones, catalyzing the intracellular conversion of the prohormone T4 into its active form T3. In addition, it was shown that DIO2 is not a target of the ERAD-system under ER stress conditions. It has been shown that T3, produced with an increase in the activity of DIO2, can accelerate the reaction of absorption of Ca^2+^ by the sarcoplasmic reticulum in cardiomyocytes, leading to their relaxation [52].

Thus, the enhancement of SELENOM, SELENOK, SELENOT, SELENOF, and DIO2 mRNA expression is most likely due to their protective anti-apoptotic properties: activation of the ERAD-system, which contributed to a decrease in proteins with incorrect folding, their antioxidant activity, and participation in the modulation of calcium signaling.

It was shown that the generation of Ca^2+^ signals by different lines of cancer cells significantly depended on the concentration of SeNP, while the amplitude of Ca^2+^ responses also grew with an increase in the SeNP concentration. It was found that A-172 and DU-145 cells responded by generating Ca^2+^ calcium signals upon application of the lowest studied concentrations of SeNPs, while MCF-7 and Caco-2 cancer cell lines were less sensitive to them. These data correlate with our results on the induction of apoptosis and a decrease in the proliferative properties of these cell lines. Indeed, it is known that changes in [Ca^2+^]i concentration, especially due to mobilization from the ER pool, can trigger apoptosis [53]. It was shown that the generation of Ca^2+^ from intracellular pools by agonists of phosphoinositol-coupled receptors or thapsigargin in drug-sensitive and drug-resistant MCF-7 was different [54]. Perhaps, in our experiments, the relative resistance of cells to lower concentrations of SeNP explains the similar nature of Ca^2+^ responses. It is known that epithelial tight junctions provide the barrier function of the intestinal mucosa. Tight junctions are multi-protein complexes consisting of transmembrane proteins and adapter proteins that interact with many other proteins, including actin-binding proteins [55,56,57]. In addition, the mechanism of synergistic destruction of tight junctions of monolayer Caco-2 cells by ethanol and acetaldehyde was demonstrated, which consisted of an increase in [Ca^2+^]i, mitochondrial oxidative stress, and the activity of Src and MLCK kinases [58]. It is possible that the different sensitivity of the studied cancer cells to the action of nanoparticles and the efficiency of calcium responses is associated with differences in the expression of calcium-transporting systems and selenoproteins. It is known that selenoproteins, the expression level of which changes in these cell lines only under high doses of SeNP, are involved in the regulation of Ca^2+^ homeostasis and ER stress [59].

## 4. Materials and Methods

### 4.1. Preparation and Characterization of SeNP

SeNP were obtained by laser ablation in liquid. The solid target was placed at the bottom of a glass cuvette under a thin layer of deionized water. In this state, the solid target was irradiated with a laser beam. The laser beam was mixed with the target using a galvanomechanical scanner TM 2D (Ateko, Moscow, Russia). Depending on the characteristics of the laser radiation, the speed and trajectory of the laser beam, it is possible to obtain colloidal solutions of selenium nanoparticles with specified geometric parameters. We used a ytterbium fiber laser (Thorlabs, Newton, NJ, USA) with a variable pulse duration (wavelength 1064 nm; laser pulse duration from 4 to 200 ns; repetition frequency 20 kHz; average power up to 20 W; pulse energy up to 1 mJ). The procedure for obtaining SeNP is described in detail elsewhere [12]. The nanoparticle size was characterized using a DC24000 analytical centrifuge (CPS Instruments, Prairieville, LA, USA). The nanoparticle concentration and hydrodynamic radius were evaluated using the Zetasizer Ultra Red Label (Malvern, UK). The morphology of the nanoparticles was studied by electron energy loss spectroscopy using a 200FE transmission electron microscope (Carl Zeiss, Jena, Germany).

**Figure 15:**
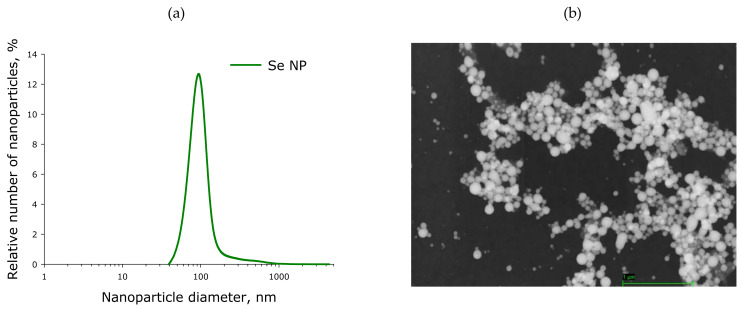
Size and morphology of SeNP obtained by laser ablation. (**a**) SeNP size distribution. Data obtained using an analytical disk centrifuge and confirmed by DLS. (**b**) TEM micrograph of selenium nanoparticles. Scale bar—1 µm.

The evolution of nanoparticle size distribution was investigated using a disk analytical centrifuge (Figure 15a). It was found that the obtained preparation of selenium nanoparticles has a monomodal size distribution. The average nanoparticle size is about 100 nm, the half-width is in the range 70–120 nm. The results obtained were confirmed by dynamic light scattering. The concentration of nanoparticles in the colloid was investigated using a Zetasizer Ultra Red Label (AZoNetwork, Manchester, UK); it was shown that, at a concentration of up to 10^13^ nanoparticles per ml, there is no aggregation in the colloidal solution. Aggregation was not detected during storage of an aqueous colloidal solution of selenium nanoparticles for a month. The zeta potential of the colloidal solution is −15.5 mV. The transmission electron microscope image shows Se nanoparticles with a diameter of about 100 nm (Figure 15b). Particle sizes shown in TEM photomicrographs matched the size distribution plotted using an analytical disk centrifuge. The shape of the nanoparticles is spherical.

### 4.2. Cell Culture and Reagents

Cell lines A-172, DU-145, MCF-7, Caco-2, and L-929 were purchased from ATCC (Manassas, VA, USA) and were harvested in a DMEM medium supplemented with 10% fetal bovine serum. MTT Cell Proliferation Assay Kit and Apoptosis/Necrosis Detection Kit were purchased from Abcam (Cambridge, UK). Antibodies for Western blots included anti-Gapdh, anti-XBP1, anti-ATF-4, anti-ATF-6, and secondary antibody conjugated to horseradish peroxidase were purchased from Abcam (Cambridge, UK) and Invitrogene (Waltham, MA, USA). Ionomycin calcium salt, Thapsigargin were purchased from Sigma-Aldrich (Saint Louis, MO, USA).

### 4.3. Cell Proliferation and Viability Assay

Viability Assay was carried on the counter Countess II FL Life Technologies (Carlsbad, CA, USA). For this, intact cells and cells with different concentrations of SeNP were seeded on a 96-well plate at 5500 cells/well and were suspended in a 1× PBS solution, stained with 0.4% trypan blue, and applied to a glass slide of the counter, where the stained cells were counted.

For cell proliferation assay, cells were seeded on a 96-well plate at 5000 cells/well and incubation was carried out for 24 h with different concentrations of SeNP. Cells were then incubated for 3 h with MTT Reagent at 37 °C. After incubation, cells were treated with MTT Solvent for 15 min at room temperature. Absorbance was measured at OD = 590 nm using Microplate reader.

### 4.4. Apoptosis/Necrosis Detection Assay

To simultaneously monitor apoptotic and healthy cells after SeNP treatment with fluorescence microscope, Apoptosis/Necrosis Detection Kit was used. Cells were seeded on a 96-well plate at 5 × 105 cells/well and incubation was carried out for 24 h with different concentrations of SeNP. Then, cells were washed 1–2 times and resuspended with Assay Buffer. To detect apoptotic cells, Apopxin Green Indicator was used. Apoptotic cells were visualized using the FITC channel (Ex/Em = 490/525 nm). For staining necrotic cells, we used 7-aminoactinomycin D (Ex/Em = 550/650 nm). To detect healthy cells, CytoCalcein 450 was used and cells were visualized using the violet channel (Ex/Em = 405/450 nm).

### 4.5. Total RNA Isolation, Reverse Transcription and Real-Time Quantitative PCR

After 24 h of SeNP treatment of cells, total RNA was isolated using the ExtractRNA reagent according to the manufacturer’s instructions Evrogene (Moscow, Russia). The concentration and purity of the total RNA were determined spectrophotometrically at 260/280 nm. First-strand cDNA was synthesized from 1–2 µg of total RNA using oligo dT primers and MMLV reverse transcriptase according to the manufacturer’s instructions Evrogene (Moscow, Russia). Quantitative Real-time PCRs were performed in a 25µL reaction mixture containing SYBR Green I PCR Master Mix Evrogene (Moscow, Russia) and 300 nM of the appropriate primers (Table 1). The PCR procedure consisted of 95 °C for 2 min followed by 40 cycles of 95 °C for 1 min, 60 °C for 30 s, and 72 °C for 30 s. Electrophoresis was performed with the PCR products to verify primer specificity and product purity. Glyceraldehyde-3-phosphate dehydrogenase (GAPDH) was used as an internal control for normalization, and results were expressed as 2-∆(∆Ct) [60].

### 4.6. Western Blot

Cells were homogenized with Cell Lysis Buffer containing 100 mM Tris-HCl, pH 8.0, 0.15 mM NaCl, 1 mM EDTA, 1 mM phenylmethanesulfonate fluoride (PMSF). The lysates were cleared by centrifugation at 14,000× *g* for 10 min at 4 °C. Proteins were separated by SDS-PAGE on 12% polyacrylamide gels and were transferred onto nitrocellulose sheets GE Healthcare (Chicago, IL, USA). Membranes were blocked for 1 h at room temperature in 5% non-fat dry milk in PBS. Nitrocellulose blots were subsequently incubated overnight at 4 °C with primary antibodies (all 1:200–1:500). Thereafter, blots were incubated for 2 h with the secondary antibody conjugated to horseradish peroxidase (1:5000). Immunoreactive bands were visualized by detection of peroxidase activity by DAB staining (0.05% DAB in TBS + 10 μL 30% hydrogen peroxide).

### 4.7. Recording Changes in Intracellular Ca^2+^

Changes in the concentration of Ca^2+^ ions in the cytosol ([Ca^2+^]i) regulates most physiological processes in health and carcinogenesis, including the induction of apoptosis.

To detect changes in [Ca^2+^]i, cancer cells were grown for 48 h in a CO_2_ incubator on round coverslips in DMEM medium supplemented with 10% fetal bovine serum until a confluence of 80–95% was achieved. Experiments on recording the level of cytosolic calcium in cells loaded with a fluorescent calcium probe Fura-2 were carried out using an image analysis system Cell observer (Carl Zeiss, Germany), based on a motorized microscope Axiovert 200 M. The experiments were carried out in Hanks solution, containing 156 mM NaCl, 3 mM KCl, 1 mM MgSO_4_, 1.25 mM KH_2_PO_4_, 2 mM CaCl_2_, 10 mM glucose, and 10 mM HEPES, pH 7.4. For excitation and registration of Fura-2 fluorescence, we used FU-2 filter set Leica (Wetzlar, Germany) with excitation filters BP340/30 and BP387/15, beam splitter FT-410, and emission filter BP510/84. Illuminator Leica EL6000 with a high-pressure mercury lamp was used as a source of excitation light.

### 4.8. Statistical Analysis

Microsoft Excel and GraphPadPrism 5 software (GraphPad, San Diego, CA, USA) was used for data analysis, graph creation, and statistic processing. The concentration of protein was calculated by standard curve plotted with 1 mg/mL of BSA solution. Values are given as the mean ± SD of at least three independent experiments. Statistical analysis was performed using a one or two-way ANOVA. In the analysis of multiple comparisons, the Sidak’s correction was used. Differences were considered significant when *p*-value was <0.05. Protein expression was quantified using ImageJ program.

## 5. Conclusions

In this work, for the first time we performed a comparative analysis of the cytotoxic effect of SeNP, obtained by laser ablation in a liquid, in four human cancer cell lines: A-172 (glioblastoma), Caco-2 (colorectal adenocarcinoma), DU-145 (prostate carcinoma), and MCF-7 (breast adenocarcinoma). It was found that cell lines A-172 and DU-145 turned out to be more sensitive to the action of lower SeNP concentrations in comparison with Caco-2 and MCF-7. Proliferative properties, viability, and the number of apoptotic cells in A-172 and DU-145 lines decreased at 0.5 μg/mL and 1 μg/mL SeNP, in contrast to the other two lines. In addition, SeNP, especially at a concentration of 5 μg/mL, had different effects on the expression patterns of the key participants of apoptosis and UPR signaling pathways, ER resident selenoproteins, and the main selenium-containing regulators of redox homeostasis in cells—thioredoxin reductases and glutathione peroxidases. For the first time, a direct correlation was shown between the sensitivity of various cancer cell lines to concentration dependent application of SeNP and the intensity of Ca^2+^ signal generation in these cells.

## Figures and Tables

**Table 1:** Primers for Real-time PCR.

Gene Name	Forward Primer 5′->3′	Reverse Primer 5′->3′
*GAPDH*	ACATCGCTCAGACACCATG	GCCAGTGAGCTTCCCGTT
*SELENOT*	TCTCCTAGTGGCGGCGTC	GTCTATATATTGGTTGAGGGAGG
*SELENOM*	AGCCTCCTGTTGCCTCCGC	AGGTCAGCGTGGTCCGAAG
*SELENOF*	TACGGTTGTTGTTGGCGAC	CAAATTGTGCTTCCTCCTGAC
*SELENOK*	TTTACATCTCGAACGGACAAG	CAGCCTTCCACTTCTTGATG
*SELENOS*	TGGGACAGCATGCAAGAAG	GCGTCCAGGTCTCCAGG
*SELENON*	TGATCTGCCTGCCCAATG	TCAGGAACTGCATGTAGGTGG
*DIO2*	AGCTTCCTCCTCGATGCC	AAAGGAGGTCAAGTGGCTG
*CHOP*	GCTCTGATTGACCGAATGG	TCTGGGAAAGGTGGGTAGTG
*GADD34*	CTCCGAGAAGGTCACTGTCC	GACGAGCGGGAAGGTGTGG
*PUMA*	CAGATATGCGCCCAGAGAT	CCATTCGTGGGTGGTCTTC
*BIM*	GGACGACCTCAACGCACAGTACGAG	GTAAGGGCAGGAGTCCCA
*ATF-4*	GTGTTCTCTGTGGGTCTGCC	GACCCTTTTCTTCCCCCTTG
*ATF-6*	AACCCTAGTGTGAGCCCTGC	GTTCAGAGCACCCTGAAGA
*XBPu*	ACTCAGACTACGTGCACCTC	GTCAATACCGCCAGAATCC
*XBPs*	CTGAGTCCGCAGCGGTGCAGG	GGTCCAAGTTGTCCAGAATG
*CAS-3*	GCATTGAGACAGACAGTGGTG	AATAGAGTTCTTTTGTGAGCATG
*CAS-4*	CACGCCTGGCTCTCATCATA	TAGCAAATGCCCTCAGCG
*MAP3K5*	AACACCTGAAGCTTAAGTCCC	TCAATGATAGCCTTCCACAGTG
*MAPK-8*	AAAGGGAACACACAATAGAAGAG	GCTGCTGCTTCTAGACTG
*BAX*	GGGCTGGACATTGGACTTC	AACACAGTCCAAGGCAGCTG
*BAK*	GAGAGTGGCATCAATTGGGG	CAGCCACCCCTCTGTGCAATCCA
*GPX1*	CTACTTATCGAGAATGTGGCG	CGAAGAGCATGAAGTTGGG
*GPX2*	CCCTTCCGACGCTACAGCCG	GGAGCCCAAGTTGAATCACC
*GPX3*	CCCCCACTCCTACTTCCTG	CCGAAGGAGCAGGGGTGG
*GPX4*	CCATGCACGAGTTTTCCG	AATTTGACGTTGTAGCCCG
*TXNRD1*	GGTCTGGCAGCTGCTAAGG	TAGCCCCAATTCAAAGAGC
*TXNRD2*	TGGGTGTGGCAGTGGGAGAC	TCCCCTGAGCCATCCCTGTG
*TXNRD3*	CCTTTGCTTTGTTGTTTCTGTG	TAGTGAGTGTGAGGGTGAAGC

## Data Availability

Not applicable.

## Abbreviations

ATF4	activating transcription factor-4
ATF6	activating transcription factor-6
BAK-BAX-BIM-BCL-2	interacting mediator of cell death
CHOP	CCAAT/enhancer-binding protein-homologous protein
ER	endoplasmic reticulum
GADD34	growth arrest and DNA damage gene-34
GPX	glutathione peroxidase
IRE1	inositol-requiring enzyme-1
MAPK3K5	mitogen-activated protein kinase kinase kinase 5
MAPK-8	mitogen-activated protein kinase 8
PERK	Protein kinase-like ER kinase
PUMA	p53 up-regulated modulator of apoptosis
SELENO	selenoprotein; SeNP-selenium nanoparticles
TXNRD	thioredoxin reductase
UPR	unfolded protein response
XBP1	X-box binding protein-1
